# Another milestone, accomplished!

**DOI:** 10.1002/jsp2.1211

**Published:** 2022-06-28

**Authors:** Mauro Alini, Robert Mauck, Daisuke Sakai

**Affiliations:** ^1^ AO Foundation, AO Research Institute Davos Switzerland; ^2^ McKay Orthopedic Research Lab University of Pennsylvania Philadelphia Pennsylvania USA; ^3^ Department of Orthopedic Surgery, Graduate School of Medicine Tokai University School of Medicine Isehara Japan

Dear Readers,

We are excited and very pleased to announce that *JOR Spine* has received its first impact factor (IF)—**3.757**! This is yet another metric that supports something we all already knew—that the *JOR Spine* is a top journal within the spine basic and translational research field!

We would like to thank all who supported and contributed, during these last 4 years, to reach this latest journal achievement, starting from the Editorial Board and the Reviewers who provided fast and rigorous reviews to the authors, to the authors themselves, who submitted their best and novel work to the journal. It was not easy, especially taking into consideration that the IF is still considered a major determinant when selecting which journal to submit and publish. We would also like to thank the ORS Spine Section for their dedication and constant support, with special issues and advertisement and encouraging their membership to support the journal. Last but not least, we are extremely grateful to the ORS and Wiley teams, for helping and guiding us during these exciting times. Thanks to all of you for the trust and commitment to make this happen, we appreciate and recognize your alliance to the journal—we would not be here without all of you!

With this new milestone, what will change now? We expect more submissions, which will imply more commitment from all of you—the Editorial Board and Reviewers—as you are driving the quality, objectivity, fairness, and speed of the review process, the most valuable deliverables for a successful journal. We also plan to involve more and more of you, by soliciting Special Issue Editors in proposing to our readers new and exciting topics within the spine research arena. Feel free to contact us to suggest your research area recommendations. We will for sure contact you!

Finally, we would like to invite you to attend this year's International Philadelphia Spine Research Symposium (6–10, November, https://www.ors.org/event/2022-ors-psrs/) where we will celebrate all together this crucial achievement and milestone for *JOR Spine* and build on this momentum. Again, thank you all for making this possible.

